# Unveiling the impact of CD133 on cell cycle regulation in radio- and chemo-resistance of cancer stem cells

**DOI:** 10.3389/fpubh.2025.1509675

**Published:** 2025-02-06

**Authors:** Luyao Wu, Takanori Katsube, Xiaofei Li, Bing Wang, Yi Xie

**Affiliations:** ^1^Institute of Modern Physics, Chinese Academy of Sciences, Lanzhou, China; ^2^Graduate School of the Chinese Academy of Sciences, Beijing, China; ^3^Institute for Radiological Science, National Institutes for Quantum Science and Technology, Chiba, Japan; ^4^Gansu Nuclear and Radiation Safety Center, Lanzhou, China

**Keywords:** malignancy, cancer stem cells, CD133, radio- and chemo-resistance, cell cycle

## Abstract

The adaptation of malignancy to therapy presents a significant challenge in cancer treatment. The cell cycle plays a crucial role in regulating the evolution of radio- and chemo-resistance in tumor cells. Cancer stem cells (CSCs) are the primary source of therapy resistance, with CD133 being one of the most recognized and valuable cell surface markers of CSCs. Evidence increasingly suggests that CD133 is associated with cancer resistance. The current understanding of the molecular biological function of CD133 is limited, leading to ongoing debates about its role in cancer biology. In this review, we explore recent research and emerging trends related to CD133 through extensive literature and content analysis. It was summarized that new insights into the relationships of CD133 and cell cycle signaling pathways in resistant CSCs. The aim of this review is to provide a foundational understanding of how these signaling pathways and their interactions impact cancer prognosis and inform treatment strategies.

## Graphical Abstract

Summary of new insights of CD133 pathways driving cell cycle regulation in cancer stem cells leading to radio- and chemotherapy resistance (By Figdraw.). 
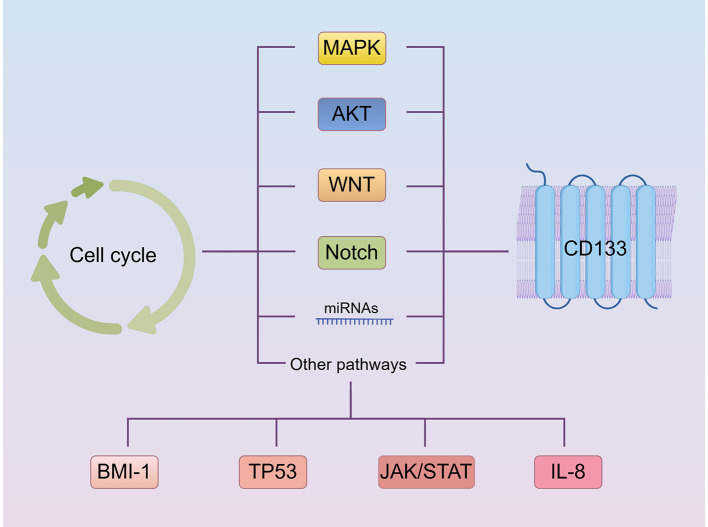

## 1 Introduction

The main reason for the high mortality rate of malignancy is the lack of effective treatments to overcome cancer radio- and chemo-resistance (1–3). Studies on the cell cycle suggest that resistance to therapy can be categorized into two types: primary resistance and acquired resistance (4, 5). Some sub-populations of tumor cells exhibit deficiencies in sensitivity to therapy depending on their specific cell cycle phase. Other sub-populations may adopt various DNA repair mechanisms or rely on faulty checkpoints to reverse or escape DNA damage by cancer therapies. Cancer stem cells (CSCs), a minority of cancer cells that can self-renew, are considered the main cause of tumor occurrence, growth, metastasis, and recurrence (6–8). CSCs inherently resist therapy due to their slow cycling, anti-apoptotic mechanism, effective DNA repair systems, and persistent stemness features (9–11). CD133 is one of the many molecules marking cancer stem cell resistance (12, 13).

CD133, or prominin-1, is a 120 kDa pentaspanning transmembrane glycoprotein, composed of five transmembrane domains, two large N-glycosylated extracellular loops, an extracellular N-terminal domain, and a cytoplasmic C-terminal domain (14). It is predominantly localized to the cell membrane, particularly within cholesterol-enriched microdomains associated with membrane protrusions (15). The extracellular N-terminal region and the two large loops are exposed to the cell exterior, whereas the C-terminal domain and two smaller loops are cytoplasmic, where they interact with the cytoskeleton to regulate cell morphology and migration (16, 17). Following glycosylation in the endoplasmic reticulum and Golgi apparatus, CD133 is integrated into the cell membrane, where it plays a crucial role in cellular signaling (18–20). Multiple transcript variants encoding distinct isoforms of this gene have been identified (21–23). Mutations in CD133 have been implicated in retinitis pigmentosa (24–26) and Stargardt disease (24, 26). It is well-known as a cell surface biomarker that allows for the recognition and sorting of stem cells from tissues or organs (27). CD133 is also a distinguishing CSC biomarkers in malignant tumors. Most CSCs markers, such as CD24, CD44, and CD133, are located in lipid rafts. CD133 is positive for CD29, CD44, CD73, and CD90. The two most important biomarkers associated with CSC are CD44 and CD133, which play important roles in diagnosis, treatment, and prognosis. However, CD133 and CD44 are differentially expressed in various solid tumors. While CD133 is often expressed in specific histological tumor types and is associated with higher tumor levels, the expression of CD44 is not related to tumor histology or tumor grading, making CD133 more clinically relevant than CD44 (28, 29).

Most research now has primarily focused on using CD133 to identify CSCs rather than understanding how CD133 operates and which pathways it may influence for optimal treatment approaches. This has sparked a debate about its role in cancer biology. Beyond its role as a reliable marker for identifying CSC populations, accumulating evidence suggests that CD133^+^ cancer cells are responsible for tumor initiation and radio- and chemo-resistance (30–32). Some studies have found higher resistance in cancer cells expressing CD133, with a growing consensus regarding the involvement of CD133 in tumor resistance. However, the exact mechanism by which above situation occurs remains unclear, with various possibilities (33, 34). It is widely accepted that cell cycle arrest can contribute to radio- and chemo-resistance. The cell cycle is the process of cell division. It is regulated by a family of proteins known as cyclin-dependent kinases (CDKs), which include CDK1, CDK2 and Cyclin dependent kinase 4/6 (CDK4/6), and cyclins (CCNs) A, B, D and E. These kinases are positively regulated by CCNs and negatively controlled by cyclin-dependent kinase inhibitors (CDKIs), such as P16, P15, p18, P19, P21, P27Kip1, and p57 (35). CDKs, their activated CCNs, and CDKIs together regulate the progression and completion of the cell cycle. Most notably, CDKs and their upstream regulatory pathways, such as PI3K/AKT, mitogen activated protein kinase (MAPK), WNT/β-catenin, Notch, and others (36–41), play key roles in the mechanism of treatment resistance (42). These transduction pathways are complex and interfere with each other (43–45). Even if the same signal transduction pathway is regulated, different tumor cells exhibit different cell cycle arrests, and the impact on CD133 or CD133 affecting them also varies.

This study examines the molecular mechanisms through which CD133 regulates the cell cycle and its contribution to radio- and chemo-resistance in cancer stem cells. A comprehensive literature search was performed using PubMed and Web of Science, employing keywords such as PIK3/AKT, MAPK, WNT/β-catenin, Notch, and miRNAs, among others. The search was restricted to publications from the past 20 years and utilized both electronic and manual retrieval methods. A total of 414 publications were identified, of which 124 were included in this systematic review. We summarize the earlier research on CD133 as they are crucial for understanding its novel roles, and describe the latest evidence proving that this unique molecule is involved in signal pathway, which affect various aspects of cellular homeostasis and cancer development.

## 2 Involvement of PI3K/AKT pathway in CD133^+^ cell cycle regulation

**Figure 1:**
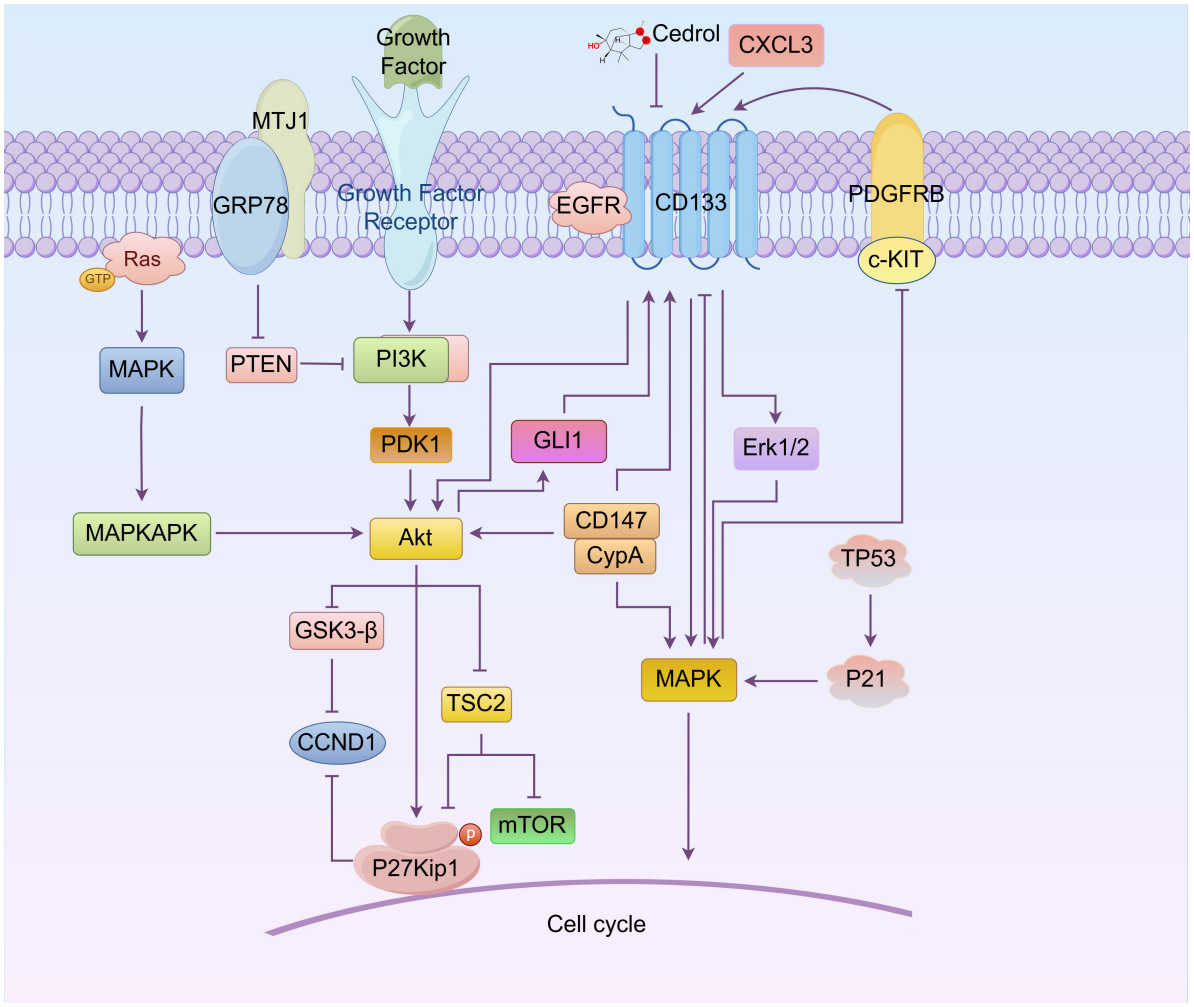
Roles of the relationship between CD133, AKT, and MAPK signaling in cell cycle regulation (By Figdraw.). CD133 directly modulates the AKT, MAPK, and ERK pathways, influencing the cell cycle progression of cancer stem cells (CSCs). AKT regulates the G_1_/S phase transition by activating downstream proteins such as mTOR, GSK3-β, CCND1, and P27Kip1, while also controlling CD133 expression through GLI1. The MAPK pathway, including key molecules such as c-KIT/PDGFRB and Erk1/2, directly or indirectly regulates CD133 expression and participates in cell cycle control. Notably, proteins such as P21 and TP53 can induce G_0_/G_1_ cell cycle arrest through MAPK signaling. Additionally, CD133 interacts with other important molecules, including GRP78, MTJ1, and EGFR, further activating PI3K/AKT and MAPK/ERK signaling pathways, which collectively influence the cell cycle.

Phosphatidylinositol-3-kinase (PI3K) is a family of proteins that catalyze transfer of the γ-phosphate of ATP to the D3 position of phosphoinositides. AKT is the primary mediator of PI3K-initiated signaling. Regarding cell cycle progression and cell growth, several targets of AKT are involved in protein synthesis, glycogen metabolism, and cell cycle regulation. As shown in Figure 1, these targets include the mammalian target of rapamycin (mTOR), glycogen synthase kinase-3 (GSK3), and the P21 and P27Kip1. AKT regulates the G_1_/S cell cycle transition through the inactivation of GSK3-β, which up-regulates Cyclin D1 (CCND1). Akt phosphorylated and inhibited tumor suppressor tuberin (TSC2), ultimately leading to a decrease in P27Kip1 (46–49). After years of research, the signaling pathways regulating the cell cycle via PI3K/AKT and mTOR are well understood.

CD133 can regulate the PI3K/Akt and MAPK pathways. Silencing CD133 suppresses PI3K/Akt and MAPK signaling pathways, leading to the downregulation of downstream targets, including RAF1, MAP2K1, MAPK3, PIK3CA, AKT3, mTOR and c-MYC, and induces cell cycle arrest at the sub-G_1_ phase (50–52). Based on this relationship, new inhibitors, arylidene derivatives, and Chinese herbal extracts have been designed to interfere with the cell cycle through these pathways, reducing the resistance of cancer stem cells. For instance, siRNA targeting midkine (a tumor promoting factor, MDK) (iMDK) (53) induces cell cycle arrest at the S and G_2_/M phases, decreases p-Akt levels, and significantly up-regulates PTEN expression in CD133-positive population of prostate cancer cells (53). Additionally, a novel arylidene derivative, IOX-101, has been shown to deactivate Akt/mTOR/NF-κB signaling and increase the sub-G_0_ phase in the cell cycle of CD133^+^ A549 cells (54). Novel derivative of matrine (MASM), suppresses the PI3K/AKT/mTOR pathway, induces cell cycle arrest at G_0_/G_1_ phase, and reduces the population of CD133^+^ cancer cells (54). Bufalin inhibits PI3K/AKT pathway, reduces the expression of multiple stemness-associated proteins, including OCT4, Sox2 and the stem cell-surface marker proteins CD133, and induces cell cycle arrest at the G_2_/M phase in gallbladder cancer cells (55).

In recent years, new pathways involving CD133 and PI3K/AKT have been discovered, as illustrated in Figure 1. For example, glucose-regulated protein 78 (GRP78), belonging to the HSP70 family, translocates and anchors at the lung cancer cell surface membrane by binding to the ER-cochaperone MTJ1. The GRP78/MTJ1 complex and CD133 are concomitantly increased in senescent H460 cells after treatment with cisplatin (DDP). The total GRP78 protein level is downregulated, while MTJ1 and downstream regulator p-AKT/AKT ratio are up-regulated (56). Additionally, CD133 physically interacts with the epidermal growth factor receptor (EGFR) in pancreatic cancer cells. CD133 appears to function as an activator of EGFR, with CD133-EGFR interaction specifically inducing the activation of PI3K/AKT downstream signaling pathways (57). Studies also show that PI3K/AKT signaling acts as an upstream signal of Glioma-associated oncogene homolog 1 (GLI1), and the upregulation of GLI1 can increase CD133 expression (58).

## 3 Understanding mitogen-activated protein kinase (MAPK) signaling pathways in CD133^+^ cell cycle regulation

The mitogen-activated protein kinase (MAPK) family in vertebrates includes p38, extracellular signal-regulated kinase (59), and c-Jun NH2-terminal kinase (JNK). Each MAPK signaling pathway consists of a MAPK kinase (MAP3K), a MAPK kinase (MAP2K), and a MAPK. These signaling pathways orchestrate numerous cellular processes, including cell growth, survival, differentiation, and apoptosis (52, 60–62). In the context of the relationship between MAPK and the cell cycle, some anticancer drugs are used to inhibit the progression of resistant cancer cells. For instance, Cedrol, a sesquiterpene alcohol, can reduce drug resistance proteins and CD133 expression in glioblastoma cells, causing cell cycle arrest at the G_0_/G_1_ phase, whereas temozolomide (TMZ)-treated cells are arrested at the G_2_/M phase. Combination treatment induces cell cycle arrest at the G_0_/G_1_ phase at 6–24 h and at the G_2_/M phase at 48 h. The expression of TP53 and P21 is increased, whereas that of CDK4, CCND1, CKD2, Cyclin A (CCNA), and Cyclin B1 (CCNB1) are decreased via regulation of the AKT and MAPK signaling pathways (63). Berberine can attenuate CD133 and potentiate G_0_/G_1_ cell cycle arrest by inhibiting PI3K/AKT and MAPK/ERK signaling and subsequently upregulating p38-MAPK in neuroblastoma cells (64) as showed in Figure 1.

The relationship between CD133 and p38-MAPK involves cell cycle regulation, as illustrated in Figure 1. Cyclophilin A (CypA), belonging to the immunophilin family, catalyzes the isomerization of peptidyl–prolyl bonds. Secreted CypA can bind to CD147, and forced down-regulation of CypA expression by natural inhibitors can inhibit CD133 expression and induce cell cycle arrest at the G_0_/G_1_ phase. This is associated with the regulation of CypA/CD147-mediated AKT and MAPK signaling pathways (65, 66). Platelet-derived growth factors (PDGFs) include five dimeric forms derived from pairs of A, B, C, and D peptide chains. PDGF dimers activate two specific receptors: PDGFRA, which binds the A, B, and C chains, and PDGFRB, which binds the B and D chains. PDGF receptors (PDGFRs) A and B are transmembrane glycoproteins that belong to the type III receptor tyrosine kinase family. This family also includes KIT, FLT3, and c-Fms1 (67). Stem cell factor is a dimeric molecule that exerts its biological functions by binding to and activating the receptor tyrosine kinase c-Kit. Data indicate that knockdown of PDGFRB and c-Kit expression in glioblastoma multiforme cells leads to inhibited CD133 expression and a normal-like cell cycle distribution driven by the MAPK/ERK signaling pathway rather than the PI3K/AKT pathway (68). MAPK can inhibit CD133 expression, whereas CD133 overexpression significantly activates Erk. The mechanism involves exogenous CXCL3, which induces Erk1/2 phosphorylation and further promotes CD133 expression. Sustained ERK signaling facilitates G_2_ cell cycle exit and primes cells for whole-genome duplication.

## 4 WNT/β-catenin signaling in CD133^+^ cell cycle regulation

**Figure 2:**
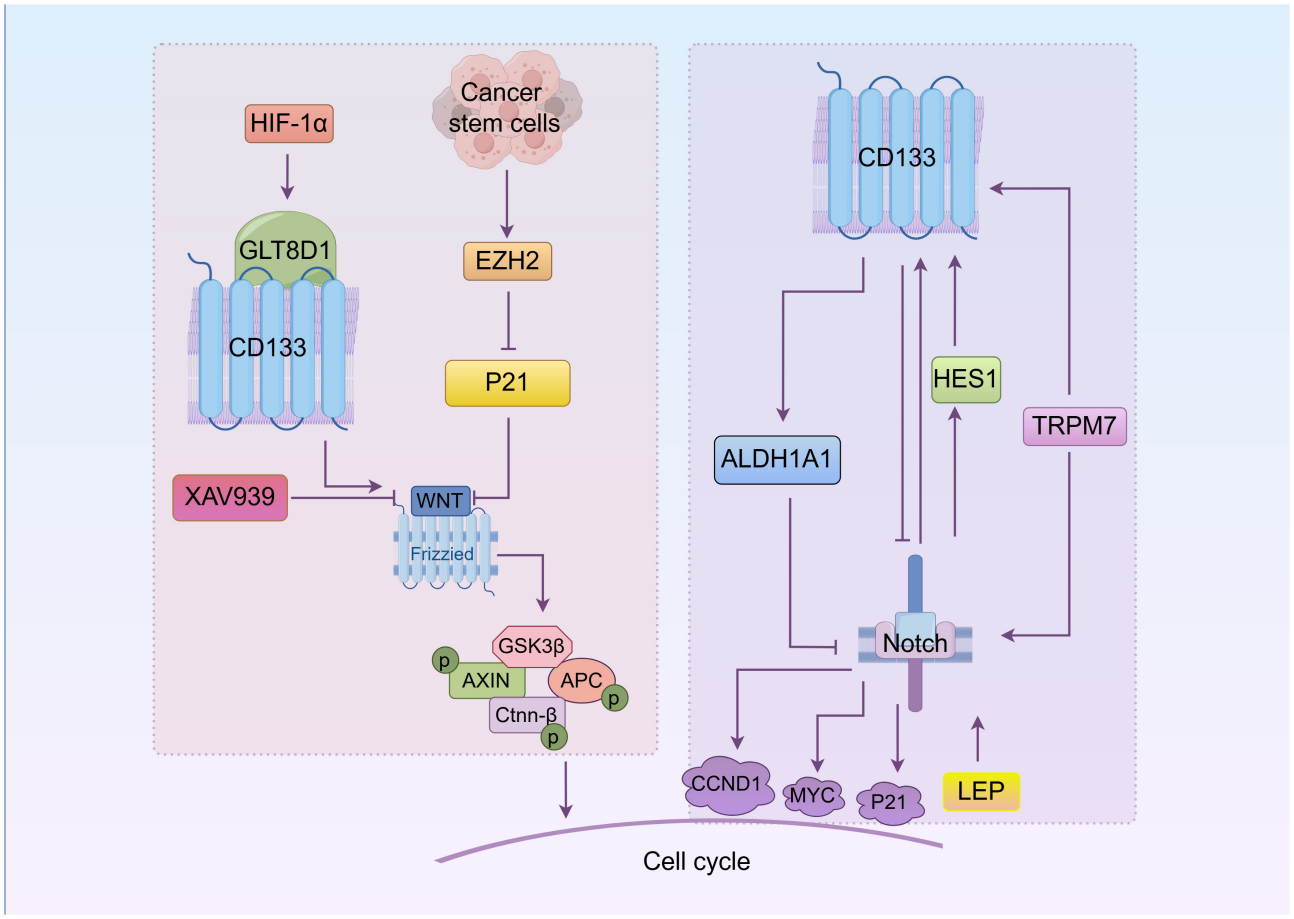
The roles of WNT and Notch in CD133^+^cell cycle regulation (By Figdraw.). CD133 directly modulates CSCs' cell cycle through the WNT/β-catenin and Notch signaling pathways. In the WNT/β-catenin pathway, EZH2 activates WNT signaling by downregulating P21, promoting the G_1_/S phase transition. Hypoxia-induced GLT8D1 binds CD133 to activate WNT signaling and drive tumorigenesis. The WNT inhibitor XAV939 reduces CD133 expression and induces G_0_/G_1_ phase arrest. In the Notch pathway, Notch regulates CD133 via HES1, influencing the cell cycle. Notch inhibition decreases CD133 expression and induces G_0_/G_1_ arrest. Notch target genes include CCND1, P21, and MYC. TRPM7 upregulates CD133 and activates Notch signaling, while leptin (LEP) enhances Notch receptor expression to promote CD133 and accelerate S phase progression. ALDH1A1 upregulation inhibits Notch signaling and promotes G_1_ arrest in lung adenocarcinoma stem cells.

WNT/β-catenin signaling is known to control many aspects of cell behavior, including a direct link with the cell cycle machinery. An aberrant WNT signaling pathway is associated with a wide array of tumor types and plays an important role in the drug resistance of CSCs (69–71). The relationship between CD133, WNT, and the cell cycle is shown in Figure 2. Enhancer of zeste homolog 2 (EZH2) is highly expressed in colorectal cancer stem-like cells. CD133 regulates the cell cycle in colorectal cancer stem-like cells by activating the WNT/β-catenin pathway via EZH2, which upregulates P21cip1 and promotes G_1_/S phase arrest. EZH2 knockdown significantly reduces the CD133^+^/CD44^+^ subpopulation (72). The upregulation of glycosyltransferase 8 domain-containing 1 (GLT8D1) in cancerous cells, induced by hypoxia or HIF-1α, is associated with more aggressive disease in gliomas. GLT8D1 knockdown promotes cell cycle arrest at the G_2_/M phase and cellular apoptosis in glioma stem cell. GLT8D1 impedes CD133 degradation through the endosomal-lysosomal pathway by N-linked glycosylation and protein-protein interactions. By directly blocking the GLT8D1/CD133 complex formation or inhibiting GLT8D1 expression, WNT/β-catenin signaling-dependent tumorigenesis is suppressed (73). The WNT signaling pathway inhibitor XAV939 significantly decreases the percentage of cells in the G_0_/G_1_ phase and increases apoptosis induced by 5-fluorouracil (5-FU)/DDP, accompanied by altered protein expression levels of β-catenin and Axin (74). Another small molecule inhibitor of WNT/β-catenin pathway, FH535, represses the expression of CD133, significantly decreasing the proportion of colon cancer cells in the S phase while increasing those in the G_0_/G_1_ phase, and effectively down-regulating target genes including CCND1 and survivin (75).

## 5 Notch/CD133 pathway

The Notch pathway is implicated in cell fate allocation and cell proliferation. Depending on the context, Notch signaling can either inhibit (76–78) or promote (79, 80) cell cycle progression. Notch target genes include key cell cycle regulators such as CCND1, P21, and MYC (81–85).

The role of Notch in CD133^+^ cell cycle is illustrated in Figure 2. Notch blockade suppresses the expression of HES1 and CD133 expression (86). Notch signaling is expressed in both CD133^+^ and CD133^−^ lung cancer cells. However, Notch1 and Notch2 are less expressed in CD133^+^ cells, causing cell cycles arrest at the G_0_/G_1_ phase spontaneously. This is because HES1 is expressed in CD133^−^ cells but not in CD133^+^ cells, indicating that the effect of Notch on CD133 is mediated by HES1 (45). Additionally, reducing the expression of transient receptor potential melastatin-related 7 (TRPM7), a non-specific divalent cation channel with a functional serine/threonine-protein kinase domain at its C-terminus, slows cells progression into certain phases of cell cycle (S and G_2_/M) and encourages apoptosis. TRPM7 expression is positively linked to Notch1 signaling activity and CD133 expression. Kinase-inactive mutants of TRPM7 result in reduced activation of Notch1 signaling and decreased CD133 expression compared to wild-type TRPM7 (87). In pancreatic cancer, leptin (LEP) increases CD133 expression, the number of cells in the S phase, and overall progression and proliferation through increased expression of Notch receptors, ligands, and target molecules (NOTCH1-4, DLL4, JAG1, BIRC5 [survivin], and HEY2). LEP regulation of Notch may be related to Ras mutations (88). In addition, elevated ALDH1A1 expression prolongs the G_1_ stage of the cell cycle and inhibits the cell cycle progression by suppressing the Notch/CDK2/CCNE1 in lung adenoma stem cells (89).

## 6 MicroRNAs (miRNAs) and long non-coding RNAs (lncRNA)

**Figure 3:**
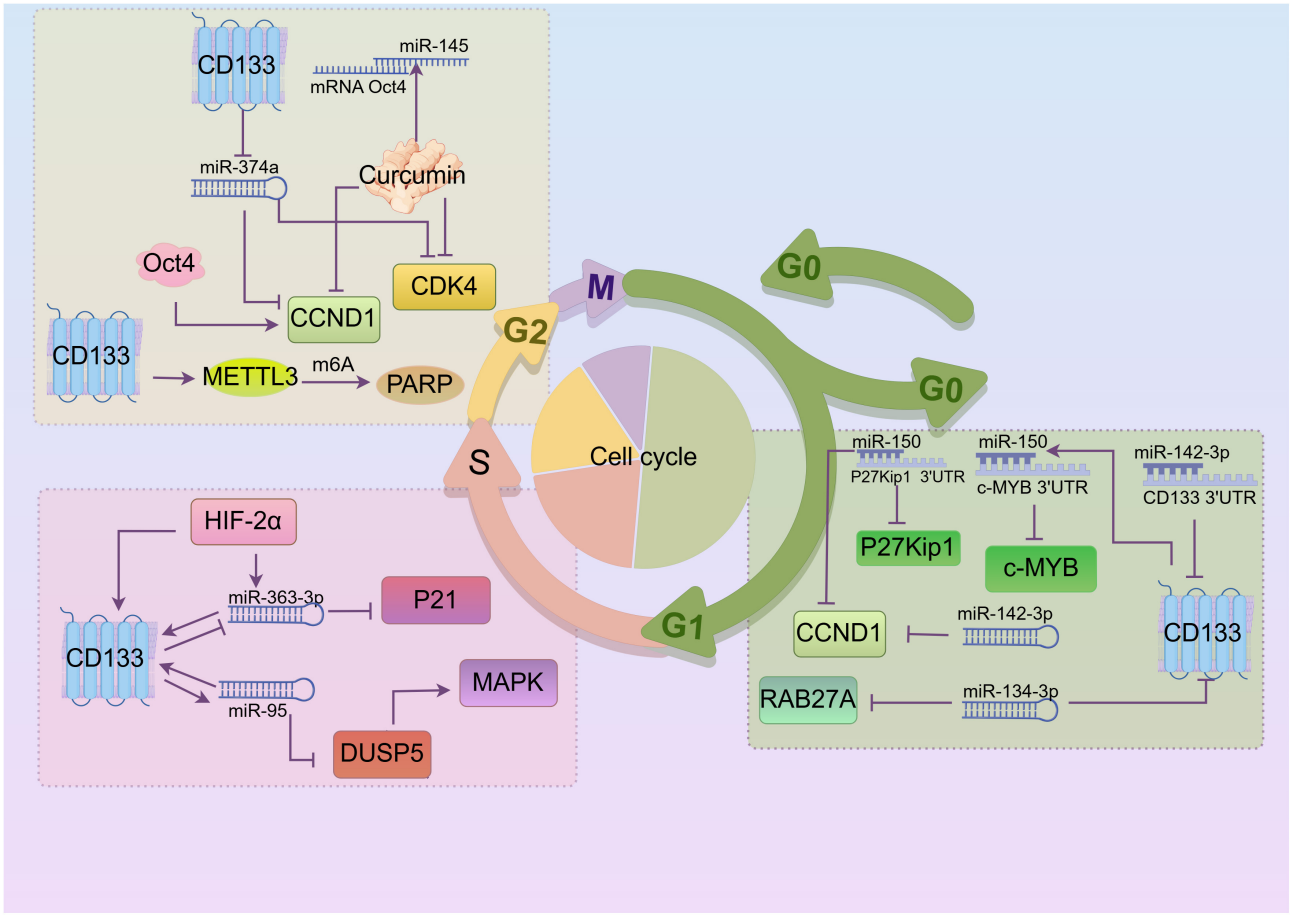
Crucial miRNAs impacting CD133 and cell cycle regulation (By Figdraw.). In the G_1_ phase, CD133 is positively correlated with miR-150, which interacts with CDKI to influence the cell cycle. miR-150 and miR-142-3p down-regulate CCND1 expression, while miR-142-3p and miR-134-3p are negatively correlated with CD133 expression. In addition, miR-150 down-regulates c-MYB protein levels, and miR-134-3p inhibits RAB27A expression. During the S phase, miR-363-3p and miR-95 regulate each other along with CD133. Overexpression of HIF-2α increases miR-363-3p expression, and miR-95 negatively regulates DUSP5. DUSP5 can activate MAPK pathway. A small amount of curcumin can suppress CCND1, CDK4, and CD133 expression, resulting in G_2_/M phase arrest. Moreover, CD133 inhibits miR-374a, which in turn suppresses CCND1 and CDK4 expression. Knocking out METTL3 caused cell cycle arrest in the G_2_/S phase in CD133^+^ cancer stem cells.

miRNAs have been implicated in maintaining the CSCs phenotype through their ability to influence the expression of genes and proteins that regulate cell proliferation and/or cell death (90–92). Crucial miRNAs impacting the cell cycle and CD133 are shown in Figure 3. miR-142-3p, miR-374a, and miR-134-3p are correlated with CD133 expression. A study has found that miR-150 expression is significantly up-regulated in CD133^+^ cancer cell subgroups (93). miR-150 interacts with the 3'UTR of c-MYB and P27^Kip1^ mRNA (93), leading to the down-regulation of c-MYB and P27^Kip1^ protein levels and inducing G_0_/G_1_ phase arrest. There is an interaction between CD133 mRNA and miR-142-3p (94). Transfection of miR-142-3p and miR-150 down-regulated CCND1 expression and induced G_1_ phase cell cycle arrest (94, 95). High expression of miR-374a down-regulated CCND1 and CDK4 protein expression, and increased the number of cells in the G_2_/M phase in CD133^+^ human glioblastoma stem cells. Additionally, Expression of miR-134-3p in human ovarian cancer stem cells not only inhibited the expression of RAB27A but also induced G_0_/G_1_ phase arrest (96, 97). The study demonstrated that miR-95 expression was significantly higher in the ALDH1^+^CD133^+^ subpopulation (98). Both miR-363-3p and miR-95 can up-regulate the level of CD133 expression, leading to induction of S phase cell cycle arrest. However, the study suggested that miR-363-3p was significantly down-regulated in CD133^+^ larynx cancer stem cells (99). Overexpression of HIF-2α significantly increased levels of miR-363-3p, and the expression of CD133 and HIF-2α is positively correlated (100). The target gene of miR-363-3p is P21, a key inhibitor of S-phase DNA synthesis and cell cycle progression (101). miR-95 negatively regulates DUSP5, inhibits MAPK pathway activation, and increases proportion of gastric cancer cells in the S phase. Curcumin treatment induces high miR-145 expression and inhibits the expression of lncRNA-ROR. Both lncRNA-ROR and Oct4 mRNA contain miR-145 binding sites and directly compete for miR-145, leading to curcumin-induced inhibition of CCND1, CDK4 and CD133 expressions, and G_2_/M phase arrest (102). The transcription factor Oct4 significantly increases wild-type CCND1 promoter activity. There are also other RNAs involved. CD133^+^ tumor stem cells exhibit up-regulated expression of N6-methyladenosine (m6A) mRNA and its writer METTL3. METTL3 enhances the stability of PARP1 by mediating m6A modification of PARP1 mRNA and recruiting YTHDF1 to its 3'-untranslated region (3'-UTR), exerting the DNA damage repair ability of PARP1. Knocking down METTL3 arrests the cell cycle in the G_2_/S phase (103, 104). The up-regulation of circRNA-ABCC1 (circ-ABCC1) contributes to the colorectal cancer cell stemness, spheroid formation, and metastasis of CD133^−^ colorectal cancer cells. The interaction between β-catenin and circ-ABCC1 can activate WNT/β-catenin and promote the progression of colorectal cancer (105).

## 7 Other pathways

**Figure 4:**
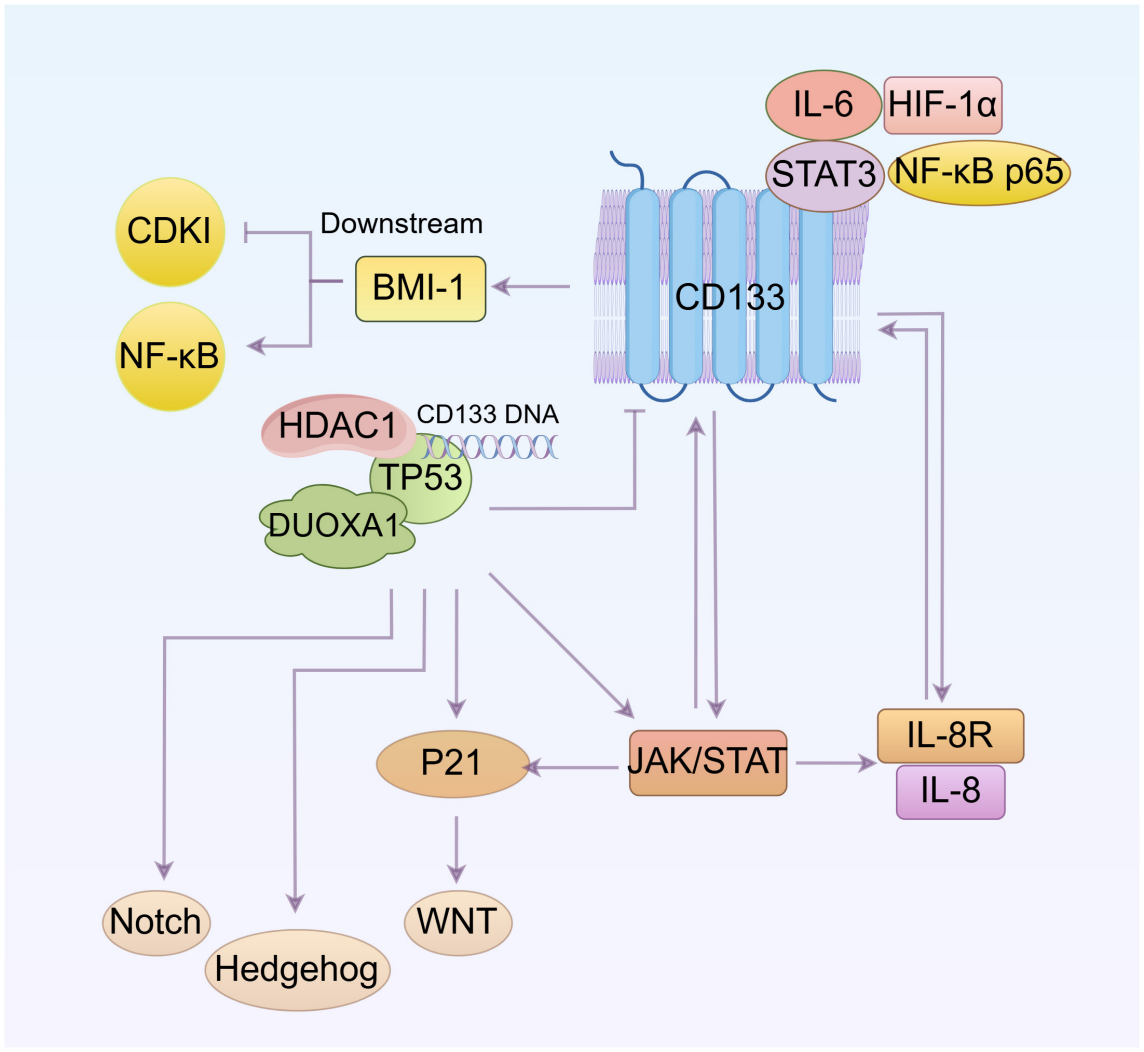
Other pathways involved in the regulation of CD133 and the cell cycle (By Figdraw.). CD133 up-regulates BMI1 expression and activates the NF-κB signaling pathway. The interaction between DUOXA1 and TP53 can influence the cell cycle by affecting the WNT, Notch, and Hedgehog pathways. TP53 suppresses CD133 expression. The IL-8 and IL-8 receptor complex, along with STAT, can induce CD133 expression, and CD133 itself can influence IL-8 levels and JAK/STAT signaling pathway. Additionally, IL-6 and STAT3 bind to the CD133 promoter, increasing CD133 protein levels. Both IL-6/STAT3 and NF-κB subunit positively regulate HIF-1α transcription. The relationship between these pathways is that NF-κB and JAK/STAT are mutually regulated, with both ways regulating IL-8. p53 can activate JAK/STAT, and P21 expression is mediated by JAK/STAT activation.

Other pathways involved in CD133 and cell cycle regulation are discussed in Figure 4.

### 7.1 BMI1

BMI1 maintains cell self-renewal and cell cycle progression, preventing cell senescence by inhibiting the transcription of the INK4a/ARF gene, which encodes a cell cycle-dependent kinase inhibitory factor (CDKI) (106). And studies have shown that BMI1 can also determines cell stemness by maintaining redox balance and preventing cell cycle arrest (107). Additionally, the data indicate that decreased CD133 expression downregulates BMI1 expression and causes cell cycle arrest at the G_0_/G_1_ and S phase by inhibiting the NF-κB signaling pathway (108).

### 7.2 TP53

The transcription factor TP53, a tumor suppressor, plays a core role in regulating the cell division cycle (109). It negatively regulates CD133 expression by binding directly to a specific sequence in the CD133 promoter. This binding recruits histone deacetylase 1 (HDAC1), which then reduces histone H3 acetylation and inhibits CD133 expression (110). There is also a notable interaction between Dual oxidase maturation factor 1 (DUOXA1) and TP53. Upregulation of TP53 significantly increases the expression of DUOXA1, which in turn reduces CD133 expression. TP53-mediated cell-cycle arrest is primarily driven by the transcriptional activation of P21 (111). Inhibition of TP53 results in cell cycle arrest of the CSC population at both the G_1_/S and G_2_/M phases, induces ROS-mediated apoptosis, and disrupts cell stemness and functions by modulating the WNT, Notch and Hedgehog pathways (112).

### 7.3 IL-6 and IL-8

Bone marrow-derived MSCs (BMSCs) secrete significantly more IL-8 after coculture with CD133^+^/CD44^+^ cells than those after coculture with CD133^−^/CD44^−^ cells. Overexpression of IL-8 was found in CD133^+^ cells (113, 114). Low expressions of IL-8 and IL-8R can effectively decrease CD133 expression and reduce the percentage of cells in the G_0_ phase (115).

STAT3, activated by IL-6, rapidly binds to the CD133 promoter and increases protein levels of CD133. IL-6/STAT3 interacts with the NF-κB subunit to positively regulate the transcription of HIF-1α, providing a mechanistic explanation on how these four oncogenes work together to increase CD133 activity. Decreased CD133 levels are associated with increased G_0_/G_1_ populations (116). The soluble receptor of IL-6 (sIL-6R) is a major factor in the maintenance and expansion of cancer stem cells. Activation of gp130 promotes the expression of the IL-6R gene on the membrane (mIL-6R) and specifically enhances sIL-6R secretion through proteolytic cleavage of mIL-6R in 5-FU pretreated CD133^+^ cells. sIL-6R could further activate gp130 on neighboring cells through a trans-signaling process, making these cells more sensitive to IL-6 (117).

### 7.4 JAK/STAT signaling

Studies have shown that JAK/STAT signaling pathway is over-activated in CD133^+^ cancer stem cells (118, 119). Cells treated with pimozide, a STAT5 inhibitor, show reduced expression of cancer stem cell marker proteins such as DCLK1, CD44, CD133, OCT4, and ABCG2. Down-regulation of START5 leads to decreased expression of CCND1 and CDK2, reduced Rb phosphorylation, and activation of Caspase-3 and PARP cleavage, resulting in sub-G_0_/G_1_ cell cycle arrest and apoptosis (120). Furthermore, there is a mutual regulation between STAT3 and NF-κB signaling, with a close interaction between them (121). JAK/STAT and NF-κB pathways regulate IL-8 production (122), while p53 activates the JAK/STAT pathway by increasing the expression levels of p-JAK and p-STAT (123). Additionally, JAK/STAT activation mediates the transcription, expression and nuclear localization of P21 (124).

## 8 Conclusions and perspectives

Therapeutic resistance remains a critical challenge in cancer treatment, largely driven by the persistence of CSCs, which exhibit intrinsic resistance to conventional therapies. Among CSC markers, CD133 has emerged as a key player, closely associated with mechanisms of therapy resistance. However, its molecular and biological functions remain poorly defined, fueling ongoing debate. Current evidence links CD133 to the regulation of cell cycle dynamics and therapy resistance, particularly in radio- and chemo-resistant tumors. Recent studies have illuminated the connection between CD133 and key signaling pathways, including PI3K/AKT, WNT/β-catenin, and Notch, which govern cell cycle checkpoints and tumor progression. CD133^+^ cells exhibit distinct mechanisms of cell cycle arrest, influenced by the genetic context of the tumor, underscoring the complexity of its role in therapy resistance. Targeting CD133 has been shown to disrupt oncogenic signaling and enhance the sensitivity of CSCs to therapeutic interventions, identifying CD133 as a compelling target for overcoming resistance. Emerging research highlights the potential of CD133-targeted therapies combined with radio- or chemotherapy to enhance treatment efficacy. By modulating the cell cycle and promoting CSC proliferation, these strategies may increase CSC susceptibility to therapeutic agents. Furthermore, novel drugs targeting CD133 and its signaling networks show promise in addressing radio-resistance, a major barrier in cancer treatment. Despite these advances, current findings remain fragmented, with unresolved questions about the interactions between CD133 and key cell cycle regulators, such as cyclins, CDKs, and checkpoint proteins. Future studies should focus on delineating the precise mechanisms by which CD133 regulates the cell cycle and contributes to therapy resistance. Such insights are vital for developing combinatorial approaches integrating CD133-targeted agents with standard treatments to overcome CSC-mediated resistance and improve therapeutic outcomes. A deeper understanding of these mechanisms will provide critical guidance for optimizing cancer treatment strategies.

## References

[1] Klein G. Evolutionary aspects of cancer resistance. Semin Cancer Biol. (2014) 25:10–4. 10.1016/j.semcancer.2014.01.001 24440448

[2] Trivedi DD Dalai SK Bakshi SR. The mystery of cancer resistance: a revelation within nature. J Mol Evol. (2023) 91:133–55. 10.1007/s00239-023-10092-6 36693985

[3] Siegel RL Giaquinto AN Jemal A. Cancer statistics, 2024. CA Cancer J Clin. (2024) 74:12–49. 10.3322/caac.21820 38230766

[4] Wu Y Song Y Wang R Wang T. Molecular mechanisms of tumor resistance to radiotherapy. Mol Cancer. (2023) 22:96. 10.1186/s12943-023-01801-2 37322433 PMC10268375

[5] Dökümcü K Farahani RM. Evolution of resistance in cancer: a cell cycle perspective. Front Oncol. (2019) 9:376. 10.3389/fonc.2019.00376 31143706 PMC6520611

[6] Knopik-Skrocka A Sempowicz A Piwocka O. Plasticity and resistance of cancer stem cells as a challenge for innovative anticancer therapies - do we know enough to overcome this? Excli J. (2024) 23:335–55. 10.17179/excli2024-6972 38655094 PMC11036066

[7] Batlle E Clevers H. Cancer stem cells revisited. Nat Med. (2017) 23:1124–34. 10.1038/nm.4409 28985214

[8] Ajmeera D Ajumeera R. Drug repurposing: a novel strategy to target cancer stem cells and therapeutic resistance. Genes Dis. (2024) 11:148–75. 10.1016/j.gendis.2022.12.013 37588226 PMC10425757

[9] Fernandes GMM Serafim Junior V Galbiatti-Dias ALS Ferreira LAM Castanhole-Nunes MMU Kawasaki-Oyama RS. Treatment effects of the Egfr pathway drugs on head and neck cancer stem cells. Am J Cancer Res. (2022) 12:4196–210. 36225637 PMC9548020

[10] Xie C Zhou X Liang C Li X Ge M Chen Y. Apatinib triggers autophagic and apoptotic cell death via Vegfr2/Stat3/Pd-L1 and Ros/Nrf2/P62 signaling in lung cancer. J Exp Clin Cancer Res. (2021) 40:266. 10.1186/s13046-021-02069-4 34429133 PMC8385858

[11] Asadzadeh Z Mansoori B Mohammadi A Kazemi T Mokhtarzadeh A Shanehbandi D. The combination effect of prominin1 (Cd133) suppression and oxaliplatin treatment in colorectal cancer therapy. Biomed Pharmacother. (2021) 137:111364. 10.1016/j.biopha.2021.111364 33592546

[12] Mare M Colarossi L Veschi V Turdo A Giuffrida D Memeo L. Cancer stem cell biomarkers predictive of radiotherapy response in rectal cancer: a systematic review. Genes. (2021) 12:101502. 10.3390/genes12101502 34680897 PMC8535834

[13] Taghizadeh-Hesary F Akbari H Bahadori M Behnam B. Targeted anti-mitochondrial therapy: the future of oncology. Genes. (2022) 13:101728. 10.3390/genes13101728 36292613 PMC9602426

[14] Corbeil D Röper K Fargeas CA Joester A Huttner WB. Prominin: A story of cholesterol, plasma membrane protrusions and human pathology. Traffic. (2001) 2:82–91. 10.1034/j.1600-0854.2001.020202.x 11247306

[15] Shmelkov SV St Clair R Lyden D Rafii S. Ac133/Cd133/Prominin-1. Int J Biochem Cell Biol. (2005) 37:715–9. 10.1016/j.biocel.2004.08.010 15694831

[16] Wang H Gong P Li J Fu Y Zhou Z Liu L. Role of Cd133 in human embryonic stem cell proliferation and teratoma formation. Stem Cell Res Ther. (2020) 11:208. 10.1186/s13287-020-01729-0 32460847 PMC7251672

[17] Sarrett SM Rodriguez C Delaney S Hosny MM Sebastiano J Santos-Coquillat A. Evaluating Cd133 as a radiotheranostic target in small-cell lung cancer. Mol Pharm. (2024) 21:1402–13. 10.1021/acs.molpharmaceut.3c01063 38331430 PMC10915790

[18] Gisina A Kim Y Yarygin K Lupatov A. Can Cd133 be regarded as a prognostic biomarker in oncology: pros and cons. Int J Mol Sci. (2023) 24:417398. 10.3390/ijms242417398 38139228 PMC10744290

[19] Immervoll H Hoem D Sakariassen P Steffensen OJ Molven A. Expression of the “stem cell marker” Cd133 in pancreas and pancreatic ductal adenocarcinomas. BMC Cancer. (2008) 8:48. 10.1186/1471-2407-8-48 18261235 PMC2268945

[20] Mak AB Pehar M Nixon AM Williams RA Uetrecht AC Puglielli L. Post-translational regulation of Cd133 by atase1/atase2-mediated lysine acetylation. J Mol Biol. (2014) 426:2175–82. 10.1016/j.jmb.2014.02.012 24556617 PMC4317256

[21] Liu Y Ren S Xie L Cui C Xing Y Liu C. Mutation of N-linked glycosylation at Asn548 in Cd133 decreases its ability to promote hepatoma cell growth. Oncotarget. (2015) 6:20650–60. 10.18632/oncotarget.4115 26029999 PMC4653032

[22] Wei Y Jiang Y Zou F Liu Y Wang S Xu N. Activation of Pi3k/Akt pathway by Cd133-P85 interaction promotes tumorigenic capacity of glioma stem cells. Proc Natl Acad Sci USA. (2013) 110:6829–34. 10.1073/pnas.1217002110 23569237 PMC3637720

[23] Kemper K Sprick MR de Bree M Scopelliti A Vermeulen L Hoek M. The Ac133 epitope, but not the Cd133 protein, is lost upon cancer stem cell differentiation. Cancer Res. (2010) 70:719–29. 10.1158/0008-5472.CAN-09-1820 20068153

[24] Puertas-Neyra K Coco-Martin RM Hernandez-Rodriguez LA Gobelli D Garcia-Ferrer Y Palma-Vecino R. Clinical exome analysis and targeted gene repair of the C.1354dupt variant in Ipsc lines from patients with prom1-related retinopathies exhibiting diverse phenotypes. Stem Cell Res Ther. (2024) 15:192. 10.1186/s13287-024-03804-2 38956727 PMC11218195

[25] Arrigoni FI Matarin M Thompson PJ Michaelides M McClements ME Redmond E. Extended extraocular phenotype of prom1 mutation in kindreds with known autosomal dominant macular dystrophy. Eur J Hum Genet. (2011) 19:131–7. 10.1038/ejhg.2010.147 20859302 PMC3025782

[26] Kim JM Lee C Lee GI Kim NKD Ki CS Park WY. Identification of the prom1 mutation PR373c in a Korean patient with autosomal dominant stargardt-like macular dystrophy. Ann Lab Med. (2017) 37:536–9. 10.3343/alm.2017.37.6.536 28840994 PMC5587829

[27] Miyasaki DM Senegaglia AC de Moura SAB Leitolis A Capriglione LGA Fracaro L. Treatment of chronic kidney disease with extracellular vesicles from mesenchymal stem cells and Cd133(+) expanded cells: a comparative preclinical analysis. Int J Mol Sci. (2022) 23:52521. 10.3390/ijms23052521 35269664 PMC8910174

[28] Tong X Xu Y Zhang T Deng C Xun J Sun D. Exosomes from Cd133(+) human urine-derived stem cells combined adhesive hydrogel facilitate rotator cuff healing by mediating bone marrow mesenchymal stem cells. J Orthopaedic Translation. (2023) 39:100–12. 10.1016/j.jot.2023.02.002 36879794 PMC9984782

[29] Singh S Bhardwaj M Sen A Nambiyar K Ahuja A. Cancer stem cell markers - Cd133 and Cd44 - in paediatric solid tumours: a study of immunophenotypic expression and correlation with clinicopathological parameters. Indian J Surg Oncol. (2023) 14:113–21. 10.1007/s13193-022-01626-3 36891437 PMC9986167

[30] Grskovic B Ruzicka K Karimi A Qujeq D Müller MM. Cell cycle analysis of the Cd133+ and Cd133- cells isolated from umbilical cord blood. Clin Chim Acta. (2004) 343:173–8. 10.1016/j.cccn.2004.01.023 15115691

[31] You CZ Xu H Zhao FS Dou J A. Validation study of Cd133 as a reliable marker for identification of colorectal cancer stem-like cells. Bull Exp Biol Med. (2024) 176:369–75. 10.1007/s10517-024-06026-x 38340198

[32] Paya L Rafat A Talebi M Aghbali A Shahidi N Nejati B. The effect of tumor resection and radiotherapy on the expression of stem cell markers (Cd44 and Cd133) in patients with squamous cell carcinoma. Int J Hematol-Oncol Stem Cell Res. (2024) 18:92–9. 10.18502/ijhoscr.v18i1.14748 38680713 PMC11055418

[33] Tatar C Avci CB Acikgoz E Oktem G. Doxorubicin-induced senescence promotes resistance to cell death by modulating genes associated with apoptotic and necrotic pathways in prostate cancer Du145 Cd133(+)/Cd44(+) cells. Biochem Biophys Res Commun. (2023) 680:194–210. 10.1016/j.bbrc.2023.09.032 37748252

[34] Dorna D Paluszczak J. Targeting cancer stem cells as a strategy for reducing chemotherapy resistance in head and neck cancers. J Cancer Res Clin Oncol. (2023) 149:13417–35. 10.1007/s00432-023-05136-9 37453969 PMC10587253

[35] da Silveira WA Renaud L Hazard ES Hardiman G. Mirna and Lncrna expression networks modulate cell cycle and DNA Repair inhibition in senescent prostate cells. Genes. (2022) 13:20208. 10.3390/genes13020208 35205253 PMC8872619

[36] Erisik D Ozdil B Acikgoz E Asker Abdikan CS Yesin TK Aktug H. Differences and similarities between colorectal cancer cells and colorectal cancer stem cells: molecular insights and implications. ACS Omega. (2023) 8:30145–57. 10.1021/acsomega.3c02681 37636966 PMC10448492

[37] Pluta AJ Studniarek C Murphy S Norbury CJ. Cyclin-dependent kinases: masters of the eukaryotic universe. Wiley Interdiscip Rev RNA. (2023) 15:e1816. 10.1002/wrna.1816 37718413 PMC10909489

[38] Milliana A Listiyana A Mutiah R Annisa R Firadusi AF Faradila VA. The Potential of eleutherine bulbosa in inducing apoptosis and inhibiting cell cycle in breast cancer: a network pharmacology approach and *in vitro* experiments. Asian Pacific J Cancer Prevent. (2023) 24:3783–94. 10.31557/APJCP.2023.24.11.3783 38019236 PMC10772747

[39] Whitaker RH Cook JG. Stress relief techniques: P38 Mapk determines the balance of cell cycle and apoptosis pathways. Biomolecules. (2021) 11:101444. 10.3390/biom11101444 34680077 PMC8533283

[40] Shimada R Ishiguro KI. Cell cycle regulation for meiosis in mammalian germ cells. J Reprod Dev. (2023) 69:139–46. 10.1262/jrd.2023-010 36927827 PMC10267585

[41] Cuervo H Muhleder S Garcia-Gonzalez I Benedito R. Notch-mediated cellular interactions between vascular cells. Curr Opin Cell Biol. (2023) 85:102254. 10.1016/j.ceb.2023.102254 37832167

[42] Bou Antoun N Chioni AM. Dysregulated signalling pathways driving anticancer drug resistance. Int J Mol Sci. (2023) 24:512222. 10.3390/ijms241512222 37569598 PMC10418675

[43] Fang Z Meng Q Xu J Wang W Zhang B Liu J. Signaling pathways in cancer-associated fibroblasts: recent advances and future perspectives. Cancer Commun. (2023) 43:3–41. 10.1002/cac2.12392 36424360 PMC9859735

[44] Katoh M Nakagama H. Fgf receptors: cancer biology and therapeutics. Med Res Rev. (2014) 34:280–300. 10.1002/med.21288 23696246

[45] Jin S Borkhuu O Bao W Yang YT. Signaling pathways in thyroid cancer and their therapeutic implications. J Clin Med Res. (2016) 8:284–96. 10.14740/jocmr2480w 26985248 PMC4780491

[46] Chang F Lee JT Navolanic PM Steelman LS Shelton JG Blalock WL. Involvement of Pi3k/Akt pathway in cell cycle progression, apoptosis, and neoplastic transformation: a target for cancer chemotherapy. Leukemia. (2003) 17:590–603. 10.1038/sj.leu.2402824 12646949

[47] Steelman LS Pohnert SC Shelton JG Franklin RA Bertrand FE McCubrey JA. Jak/Stat, Raf/Mek/Erk, Pi3k/Akt and Bcr-Abl in cell cycle progression and leukemogenesis. Leukemia. (2004) 18:189–218. 10.1038/sj.leu.2403241 14737178

[48] Porta C Paglino C Mosca A. Targeting Pi3k/Akt/Mtor signaling in cancer. Front Oncol. (2014) 4:64. 10.3389/fonc.2014.00064 24782981 PMC3995050

[49] Tewari D Patni P Bishayee A Sah AN Bishayee A. Natural products targeting the Pi3k-Akt-Mtor signaling pathway in cancer: a novel therapeutic strategy. Semin Cancer Biol. (2022) 80:1–17. 10.1016/j.semcancer.2019.12.008 31866476

[50] Aghajani M Mokhtarzadeh A Aghebati-Maleki L Mansoori B Mohammadi A Safaei S. Cd133 suppression increases the sensitivity of prostate cancer cells to paclitaxel. Mol Biol Rep. (2020) 47:3691–703. 10.1007/s11033-020-05411-9 32246247

[51] Wu Z Li W Tang Q Huang L Zhan Z Li Y. A novel aniline derivative from Peganum Harmala L. promoted apoptosis via activating Pi3k/Akt/Mtor-mediated autophagy in non-small cell lung cancer cells. Int J Mol Sci. (2023) 24:12626. 10.3390/ijms241612626 37628807 PMC10454575

[52] Abdoli Shadbad M Nejadi Orang F Baradaran B. Cd133 significance in glioblastoma development: *in silico* and *in vitro* study. Eur J Med Res. (2024) 29:154. 10.1186/s40001-024-01754-2 38448914 PMC10918901

[53] Erdogan S Doganlar ZB Doganlar O Turkekul K Serttas R. Inhibition of midkine suppresses prostate cancer Cd133(+) stem cell growth and migration. Am J Med Sci. (2017) 354:299–309. 10.1016/j.amjms.2017.04.019 28918838

[54] Liu Y Qi Y Bai ZH Ni CX Ren QH Xu WH. A novel matrine derivate inhibits differentiated human hepatoma cells and hepatic cancer stem-like cells by suppressing Pi3k/Akt signaling pathways. Acta Pharmacol Sin. (2017) 38:120–32. 10.1038/aps.2016.104 27773936 PMC5220537

[55] Qian L Su H Wang G Li B Shen G Gao Q. Anti-tumor activity of bufalin by inhibiting C-Met mediated Mek/Erk and Pi3k/Akt signaling pathways in gallbladder cancer. J Cancer. (2020) 11:3114–23. 10.7150/jca.38393 32231716 PMC7097950

[56] Sriratanasak N Chunhacha P Ei ZZ Chanvorachote P. Cisplatin induces senescent lung cancer cell-mediated stemness induction via Grp78/Akt-dependent mechanism. Biomedicines. (2022) 10:2703. 10.3390/biomedicines10112703 36359223 PMC9687146

[57] Weng CC Kuo KK Su HT Hsiao PJ Chen YW Wu DC. Pancreatic tumor progression associated with Cd133 overexpression: involvement of increased tert expression and epidermal growth factor receptor-dependent Akt activation. Pancreas. (2016) 45:443–57. 10.1097/MPA.0000000000000460 26646272

[58] Yang Z Zhang C Qi W Cui Y Xuan Y. Gli1 promotes cancer stemness through intracellular signaling pathway Pi3k/Akt/Nfκb in colorectal adenocarcinoma. Exp Cell Res. (2018) 373:145–54. 10.1016/j.yexcr.2018.10.006 30321514

[59] Chang JY Senan S Paul MA Mehran RJ Louie AV Balter P. Stereotactic ablative radiotherapy versus lobectomy for operable stage I non-small-cell lung cancer: a pooled analysis of two randomised trials. Lancet Oncol. (2015) 16:630–7. 10.1016/S1470-2045(15)70168-3 25981812 PMC4489408

[60] Garcia-Flores N Jimenez-Suarez J Garnes-Garcia C Fernandez-Aroca DM Sabater S Andres I. P38 Mapk and radiotherapy: foes or friends? Cancers. (2023) 15:30861. 10.3390/cancers15030861 36765819 PMC9913882

[61] Ganguly P Macleod T Wong C Harland M McGonagle D. Revisiting P38 mitogen-activated protein kinases (Mapk) in inflammatory arthritis: a narrative of the emergence of Mapk-activated protein kinase inhibitors (Mk2i). Pharmaceuticals (Basel, Switzerland). (2023) 16:91286. 10.3390/ph16091286 37765094 PMC10537904

[62] Saleem S. Targeting Mapk Signaling: A promising approach for treating inflammatory lung disease. Pathol Res Pract. (2024) 254:155122. 10.1016/j.prp.2024.155122 38246034

[63] Chang KF Huang XF Chang JT Huang YC Lo WS Hsiao CY. Sesquiterpene alcohol, enhances the anticancer efficacy of temozolomide in attenuating drug resistance via regulation of the DNA damage response and Mgmt expression. J Nat Prod. (2020) 83:3021–9. 10.1021/acs.jnatprod.0c00580 32960603

[64] Naveen CR Gaikwad S Agrawal-Rajput R. Berberine induces neuronal differentiation through inhibition of cancer stemness and epithelial-mesenchymal transition in neuroblastoma cells. Phytomed Int J Phytother Phytopharmacol. (2016) 23:736–44. 10.1016/j.phymed.2016.03.013 27235712

[65] Han JM Jung HJ. Cyclophilin a/Cd147 interaction: a promising target for anticancer therapy. Int J Mol Sci. (2022) 23:69341. 10.3390/ijms23169341 36012604 PMC9408992

[66] Cho HJ Jung HJ. Cyclophilin a inhibitors suppress proliferation and induce apoptosis of Mkn45 gastric cancer stem-like cells by regulating Cypa/Cd147-mediated signaling pathway. Int J Mol Sci. (2023) 24:4734. 10.3390/ijms24054734 36902161 PMC10003193

[67] Vega F Medeiros LJ Bueso-Ramos CE Arboleda P Miranda RN. Hematolymphoid neoplasms associated with rearrangements of Pdgfra, Pdgfrb, and Fgfr1. Am J Clin Pathol. (2015) 144:377–92. 10.1309/AJCPMORR5Z2IKCEM 26276769

[68] Dong Y Han Q Zou Y Deng Z Lu X Wang X. Long-term exposure to imatinib reduced cancer stem cell ability through induction of cell differentiation via activation of Mapk signaling in glioblastoma cells. Mol Cell Biochem. (2012) 370:89–102. 10.1007/s11010-012-1401-0 22829019

[69] Davidson G. The cell cycle and Wnt. Cell Cycle. (2010) 9:1667–8. 10.4161/cc.9.9.11595 20404508

[70] Rasmussen ML Ortolano NA Romero-Morales AI Gama V. Wnt signaling and its impact on mitochondrial and cell cycle dynamics in pluripotent stem cells. Genes. (2018) 9:20109. 10.3390/genes9020109 29463061 PMC5852605

[71] Hayat R Manzoor M Hussain A. Wnt signaling pathway: a comprehensive review. Cell Biol Int. (2022) 46:863–77. 10.1002/cbin.11797 35297539

[72] Chen JF Luo X Xiang LS Li HT Zha L Li N. Ezh2 promotes colorectal cancer stem-like cell expansion by activating P21cip1-Wnt/?-catenin signaling. Oncotarget. (2016) 7:41540–58. 10.18632/oncotarget.9236 27172794 PMC5173077

[73] Liu K Jiang L Shi Y Liu B He Y Shen Q. Hypoxia-induced Glt8d1 promotes glioma stem cell maintenance by inhibiting Cd133 degradation through N-linked glycosylation. Cell Death Differ. (2022) 29:1834–49. 10.1038/s41418-022-00969-2 35301431 PMC9433395

[74] Wu X Luo F Li J Zhong X Liu K. Tankyrase 1 Inhibitior Xav939 increases chemosensitivity in colon cancer cell lines via inhibition of the Wnt signaling pathway. Int J Oncol. (2016) 48:1333–40. 10.3892/ijo.2016.3360 26820603 PMC4777596

[75] Chen Y Rao X Huang K Jiang X Wang H Teng L. Fh535 Inhibits proliferation and motility of colon cancer cells by targeting Wnt/Beta-catenin signaling pathway. J Cancer. (2017) 8:3142–53. 10.7150/jca.19273 29158786 PMC5665030

[76] Fang JS Coon BG Gillis N Chen Z Qiu J Chittenden TW. Shear-induced notch-Cx37-P27 axis arrests endothelial cell cycle to enable arterial specification. Nat Commun. (2017) 8:2149. 10.1038/s41467-017-01742-7 29247167 PMC5732288

[77] Alhashem Z Feldner-Busztin D Revell C Alvarez-Garcillan Portillo M Camargo-Sosa K Richardson J. Notch controls the cell cycle to define leader versus follower identities during collective cell migration. eLife. (2022) 11:73550. 10.7554/eLife.73550.sa2 35438077 PMC9129880

[78] Nicoli S Knyphausen CP Zhu LJ Lakshmanan A Lawson ND. Mir-221 is required for endothelial tip cell behaviors during vascular development. Dev Cell. (2012) 22:418–29. 10.1016/j.devcel.2012.01.008 22340502 PMC3285411

[79] Patel J Wong HY Wang W Alexis J Shafiee A Stevenson AJ. Self-renewal and high proliferative colony forming capacity of late-outgrowth endothelial progenitors is regulated by cyclin-dependent kinase inhibitors driven by notch signaling. Stem Cells. (2016) 34:902–12. 10.1002/stem.2262 26732848

[80] Zalc A Hayashi S Auradé F Bröhl D Chang T Mademtzoglou D. Antagonistic regulation of P57kip2 by Hes/Hey downstream of notch signaling and muscle regulatory factors regulates skeletal muscle growth arrest. Development (Cambridge, England). (2014) 141:2780–90. 10.1242/dev.110155 25005473

[81] Campa VM Gutiérrez-Lanza R Cerignoli F Díaz-Trelles R Nelson B Tsuji T. Notch activates cell cycle reentry and progression in quiescent cardiomyocytes. J Cell Biol. (2008) 183:129–41. 10.1083/jcb.200806104 18838555 PMC2557048

[82] Guo D Ye J Dai J Li L Chen F Ma D. Notch-1 regulates Akt signaling pathway and the expression of cell cycle regulatory proteins cyclin D1, Cdk2 and P21 in T-all cell lines. Leuk Res. (2009) 33:678–85. 10.1016/j.leukres.2008.10.026 19091404

[83] Joshi I Minter LM Telfer J Demarest RM Capobianco AJ Aster JC. Notch signaling mediates G1/S cell-cycle progression in T cells via cyclin D3 and its dependent kinases. Blood. (2009) 113:1689–98. 10.1182/blood-2008-03-147967 19001083 PMC2647664

[84] Palomero T Lim WK Odom DT Sulis ML Real PJ Margolin A. Notch1 directly regulates C-Myc and activates a feed-forward-loop transcriptional network promoting leukemic cell growth. Proc Natl Acad Sci USA. (2006) 103:18261–6. 10.1073/pnas.0606108103 17114293 PMC1838740

[85] Ronchini C Capobianco AJ. Induction of Cyclin D1 Transcription and Cdk2 activity by Notch(Ic): implication for cell cycle disruption in transformation by Notch(Ic). Mol Cell Biol. (2001) 21:5925–34. 10.1128/MCB.21.17.5925-5934.2001 11486031 PMC87311

[86] Fan X Matsui W Khaki L Stearns D Chun J Li YM. Notch pathway inhibition depletes stem-like cells and blocks engraftment in embryonal brain tumors. Cancer Res. (2006) 66:7445–52. 10.1158/0008-5472.CAN-06-0858 16885340

[87] Wan J Guo AA King P Guo S Saafir T Jiang Y. Trpm7 induces tumorigenesis and stemness through notch activation in glioma. Front Pharmacol. (2020) 11:590723. 10.3389/fphar.2020.590723 33381038 PMC7768084

[88] Harbuzariu A Rampoldi A Daley-Brown DS Candelaria P Harmon TL Lipsey CC. Leptin-notch signaling axis is involved in pancreatic cancer progression. Oncotarget. (2017) 8:7740–52. 10.18632/oncotarget.13946 27999190 PMC5352357

[89] Li Z Xiang Y Xiang L Xiao Y Li F Hao P. Aldh maintains the stemness of lung adenoma stem cells by suppressing the Notch/Cdk2/Ccne pathway. PLoS ONE. (2014) 9:e92669. 10.1371/journal.pone.0092669 24671051 PMC3966794

[90] Lou W Liu J Gao Y Zhong G Ding B Xu L. MicroRNA regulation of liver cancer stem cells. Am J Cancer Res. (2018) 8:1126–41. 30094089 PMC6079154

[91] Li L Xun C Yu CH. Role of microRNA-regulated cancer stem cells in recurrent hepatocellular carcinoma. World J Hepatol. (2022) 14:1985–96. 10.4254/wjh.v14.i12.1985 36618329 PMC9813843

[92] Niu T Zhang W Xiao W. MicroRNA regulation of cancer stem cells in the pathogenesis of breast cancer. Cancer Cell Int. (2021) 21:31. 10.1186/s12935-020-01716-8 33413418 PMC7792222

[93] Liu DZ Zhang HY Long XL Zou SL Zhang XY Han GY. Mir-150 promotes prostate cancer stem cell development via suppressing P27kip1. Eur Rev Med Pharmacol Sci. (2015) 19:4344–52. 26636522

[94] Shen WW Zeng Z Zhu WX Fu GH. Mir-142-3p functions as a tumor suppressor by targeting Cd133, Abcg2, and Lgr5 in colon cancer cells. J Mol Med. (2013) 91:989–1000. 10.1007/s00109-013-1037-x 23619912

[95] Zhang J Luo N Luo Y Peng Z Zhang T Li S. microRNA-150 inhibits human Cd133-positive liver cancer stem cells through negative regulation of the transcription factor C-Myb. Int J Oncol. (2012) 40:747–56. 10.3892/ijo.2011.1242 22025269

[96] Pan Z Shi Z Wei H Sun F Song J Huang Y. Magnetofection based on superparamagnetic iron oxide nanoparticles weakens glioma stem cell proliferation and invasion by mediating high expression of MicroRNA-374a. J Cancer. (2016) 7:1487–96. 10.7150/jca.15515 27471565 PMC4964133

[97] Chang C Liu T Huang Y Qin W Yang H Chen J. MicroRNA-134-3p is a novel potential inhibitor of human ovarian cancer stem cells by targeting Rab27a. Gene. (2017) 605:99–107. 10.1016/j.gene.2016.12.030 28043921

[98] Du M Zhuang Y Tan P Yu Z Zhang X Wang A. microRNA-95 knockdown inhibits epithelial-mesenchymal transition and cancer stem cell phenotype in gastric cancer cells through Mapk pathway by upregulating Dusp5. J Cell Physiol. (2020) 235:944–56. 10.1002/jcp.29010 31309567

[99] Karatas OF Suer I Yuceturk B Yilmaz M Oz B Guven G. Identification of microRNA profile specific to cancer stem-like cells directly isolated from human larynx cancer specimens. BMC Cancer. (2016) 16:853. 10.1186/s12885-016-2863-3 27816053 PMC5097853

[100] Gao Y Feng J Wu L Zhan S Sun J. [Expression and pathological mechanism of Mmp-9 and Hif-2α in Cd133(+) lung cancer stem cells]. Zhonghua yi xue za zhi. (2015) 95:2607–11. 26711609

[101] Hao T Li CX Ding XY Xing XJ. MicroRNA-363-3p/P21(Cip1/Waf1) axis is regulated by Hif-2α in mediating stemness of melanoma cells. Neoplasma. (2019) 66:427–36. 10.4149/neo_2018_180828N655 30784290

[102] Liu T Chi H Chen J Chen C Huang Y Xi H. Curcumin suppresses proliferation and *in vitro* invasion of human prostate cancer stem cells by cerna effect of Mir-145 and Lncrna-Ror. Gene. (2017) 631:29–38. 10.1016/j.gene.2017.08.008 28843521

[103] Li H Wang C Lan L Yan L Li W Evans I. Mettl3 promotes oxaliplatin resistance of gastric cancer Cd133+ stem cells by promoting Parp1 Mrna stability. Cell Mol Life Sci. (2022) 79:135. 10.1007/s00018-022-04129-0 35179655 PMC11072755

[104] Ostrowska K Rawłuszko-Wieczorek AA Ostapowicz J Suchorska WM Golusiński W. The two-faced role of RNA methyltransferase Mettl3 on cellular response to cisplatin in head and neck squamous cell carcinoma *in vitro* model. Front Oncol. (2024) 14:1402126. 10.3389/fonc.2024.1402126 38966069 PMC11223524

[105] Zhao H Chen S Fu Q. Exosomes from Cd133(+) cells carrying circ-Abcc1 mediate cell stemness and metastasis in colorectal cancer. J Cell Biochem. (2020) 121:3286–97. 10.1002/jcb.29600 31960989

[106] Sahasrabuddhe AA. Bmi1: a biomarker of hematologic malignancies. Biomark Cancer. (2016) 8:65–75. 10.4137/BIC.S33376 27168727 PMC4859448

[107] Zhou J Chen A Wang Z Zhang J Chen H Zhang H. Bmi-1 determines the stemness of renal stem or progenitor cells. Biochem Biophys Res Commun. (2020) 529:1165–72. 10.1016/j.bbrc.2020.06.140 32819581

[108] Ma DQ Zhang YH Ding DP Li J Chen LL Tian YY. Effect of Bmi-1-mediated Nf-?b signaling pathway on the stem-like properties of Cd133+ human liver cancer cells. Cancer Biomark Section A Dis Mark. (2018) 22:575–85. 10.3233/CBM-181329 29843222 PMC13078480

[109] Engeland K. Cell cycle regulation: P53-P21-Rb signaling. Cell Death Differ. (2022) 29:946–60. 10.1038/s41418-022-00988-z 35361964 PMC9090780

[110] Park EK Lee JC Park JW Bang SY Yi SA Kim BK. Transcriptional repression of cancer stem cell marker Cd133 by tumor suppressor P53. Cell Death Dis. (2015) 6:e1964. 10.1038/cddis.2015.313 26539911 PMC4670923

[111] Chen J. The cell-cycle arrest and apoptotic functions of P53 in tumor initiation and progression. Cold Spring Harb Perspect Med. (2016) 6:a026104. 10.1101/cshperspect.a026104 26931810 PMC4772082

[112] Ostrakhovitch EA Semenikhin OA. P53-mediated regulation of neuronal differentiation via regulation of dual oxidase maturation factor 1. Neurosci Lett. (2011) 494:80–5. 10.1016/j.neulet.2011.02.061 21362455

[113] Tang KH Ma S Lee TK Chan YP Kwan PS Tong CM. Cd133(+) Liver tumor-initiating cells promote tumor angiogenesis, growth, and self-renewal through neurotensin/interleukin-8/Cxcl1 signaling. Hepatology (Baltimore, Md). (2012) 55:807–20. 10.1002/hep.24739 21994122

[114] Barcelos LS Duplaa C Kränkel N Graiani G Invernici G Katare R. Human Cd133+ progenitor cells promote the healing of diabetic ischemic ulcers by paracrine stimulation of angiogenesis and activation of Wnt signaling. Circ Res. (2009) 104:1095–102. 10.1161/CIRCRESAHA.108.192138 19342601 PMC2821014

[115] Ma X Chen J Liu J Xu B Liang X Yang X. Il-8/Cxcr2 Mediates tropism of human bone marrow-derived mesenchymal stem cells toward Cd133(+) /Cd44(+) colon cancer stem cells. J Cell Physiol. (2021) 236:3114–28. 10.1002/jcp.30080 33078417

[116] Won C Kim BH Yi EH Choi KJ Kim EK Jeong JM. Signal transducer and activator of transcription 3-mediated Cd133 up-regulation contributes to promotion of hepatocellular carcinoma. Hepatology (Baltimore, Md). (2015) 62:1160–73. 10.1002/hep.27968 26154152 PMC5049669

[117] Campard D Vasse M Rose-John S Poyer F Lamacz M Vannier JP. Multilevel regulation of Il-6r by Il-6-Sil-6r fusion protein according to the primitiveness of peripheral blood-derived Cd133+ cells. Stem Cells (Dayton, Ohio). (2006) 24:1302–14. 10.1634/stemcells.2005-0173 16357344

[118] Natsume A Kinjo S Yuki K Kato T Ohno M Motomura K. Glioma-initiating cells and molecular pathology: implications for therapy. Brain Tumor Pathol. (2011) 28:1–12. 10.1007/s10014-010-0011-3 21274750

[119] Zhao Q Zong H Zhu P Su C Tang W Chen Z. Crosstalk between colorectal Cscs and immune cells in tumorigenesis, and strategies for targeting colorectal Cscs. Exp Hematol Oncol. (2024) 13:6. 10.1186/s40164-024-00474-x 38254219 PMC10802076

[120] Subramaniam D Angulo P Ponnurangam S Dandawate P Ramamoorthy P Srinivasan P. Suppressing Stat5 signaling affects osteosarcoma growth and stemness. Cell Death Dis. (2020) 11:149. 10.1038/s41419-020-2335-1 32094348 PMC7039889

[121] Guo Q Jin Y Chen X Ye X Shen X Lin M. Nf-Kappab in biology and targeted therapy: new insights and translational implications. Signal Trans Target Ther. (2024) 9:53. 10.1038/s41392-024-01757-9 38433280 PMC10910037

[122] Zhou J Sun X Zhang J Yang Y Chen D Cao J. Il-34 Regulates Il-6 and Il-8 production in human lung fibroblasts via Mapk, Pi3k-Akt, Jak and Nf-?b signaling pathways. Int Immunopharmacol. (2018) 61:119–25. 10.1016/j.intimp.2018.05.023 29857241

[123] Bao D Zhuang C Jiao Y Yang L. The possible involvement of Circrna Dmnt1/P53/Jak/Stat in gestational diabetes mellitus and preeclampsia. Cell Death Disc. (2022) 8:121. 10.1038/s41420-022-00913-w 35296654 PMC8927128

[124] Lui AJ Geanes ES Ogony J Behbod F Marquess J Valdez K. Ifitm1 suppression blocks proliferation and invasion of aromatase inhibitor-resistant breast cancer *in vivo* by Jak/Stat-mediated induction of P21. Cancer Lett. (2017) 399:29–43. 10.1016/j.canlet.2017.04.005 28411130 PMC5530765

